# Lactic acidosis, a potential toxicity from drug–drug interaction related to concomitant ribociclib and metformin in preexisting renal insufficiency: A case report

**DOI:** 10.1002/cnr2.1575

**Published:** 2021-11-05

**Authors:** Chalita Lagampan, Nattaya Poovorawan, Napa Parinyanitikul

**Affiliations:** ^1^ Division of Medical Oncology, Department of Medicine, Faculty of Medicine King Chulalongkorn Memorial Hospital and Chulalongkorn University Bangkok Thailand

**Keywords:** concomitant, drug–drug interaction, lactic acidosis, renal insufficiency

## Abstract

**Background:**

Ribociclib, one of the cyclin‐dependent kinases (CDK) 4 and 6 inhibitors, in combination with endocrine therapies has been approved in the treatment of hormonal receptor positive, HER‐2 negative metastatic breast cancer worldwide. Long‐term usage of ribociclib with concomitant drugs, potential drug–drug interaction may develop which can limit the therapeutic value of CDK4/6 inhibitor.

**Case:**

A 62‐year‐old with history of non‐insulin dependent diabetic, dyslipidemia, and essential hypertension was diagnosed with HR‐positive, HER‐2 negative metastatic breast cancer and treated with fulvestrant plus ribociclib. Four weeks after administration, elevated serum creatinine was observed, and then severe lactic acidosis with acute respiratory failure was subsequently reported. Ribociclib and fulvestrant were temporarily discontinued. Three days after renal replacement therapy, her clinical was stabilized. Combination ribociclib with metformin resulted in high plasma metformin levels and dangerous consequences. Hence, special precaution should be considered during concomitant treatment with sensitive transporter substrates.

**Conclusion:**

Metformin associated lactic acidosis may potentially occur after combination with ribocilib, an uncommon but lethal complication from the interaction of these drugs, especially in patients who had preexisting renal impairment.

## 1 INTRODUCTION

Cyclin‐dependent kinase 4 and 6 inhibitors (CDK4/6), one of the significant milestone treatments have been broadly approved for the treatment of hormone‐receptor‐positive advanced breast cancer in the first, second, or later‐line settings. Adding palbociclib, ribociclib or abemaciclib with endocrine therapy have consistent significantly improved progression‐free survival and recently overall survival compared to endocrine therapy alone by several months regardless of line of treatment, menopausal status and visceral or bone metastases. All CDK4/6 inhibitors are generally well tolerated; neutropenia but not febrile neutropenia, fatigue, and nausea are the most common adverse events.^1^, ^2^, ^3^ Moreover, patient‐reported outcome (PRO) from MONALEESA‐3 demonstrated that a combination of ribociclib and fulvestrant maintains the quality of life (QoL) compared with fulvestrant plus placebo. Besides, a clinically meaningful improvement from baseline in pain score was observed in the ribociclib group.^4^, ^5^

Not only long‐term usage of ribociclib but also the problem of polypharmacy in the advanced breast cancer setting, potential drug–drug interactions (DDIs) may occur which can limit the therapeutic value of CDK4/6 inhibitor. Unlike palbociclib, ribociclib with a dose of 600 mg is a strong CYP3A4 inhibitor. Therefore, concomitant drugs either CYP3A4 inhibitors or CYP3A4 inducers that regularly prescribed in daily practice should be cautious. These interactions possibly will affect drug metabolisms which influence the efficacy and adverse toxicities. Additionally, previous in vitro study indicated that ribociclib has the potential to inhibit the activities of several drug transporters such as P‐glycoprotein (P‐gp), breast cancer resistance protein (BCRP), organic anion‐transporting polypeptide (OATP1B1/1B3), organic cationic transporter‐1 and ‐2 (OCT1, OCT2), multidrug and toxin extrusion protein‐1 (MATE1), and bile salt export pump (BSEP). However, only BCRP, OCT2, MATE1 and BSEP were inhibited at clinically relevant concentration.^6^ Metformin, one of widely used oral hypoglycemic drugs, is generally excreted unchanged into urine via the renal transporters MATE1, MATE2‐K, and OCT2. Therefore, inhibition of these transporters by ribociclib while metformin administers concomitantly can lead to increased metformin exposure and may deepens the harmful side effect of metformin.^7^

Here, we reported clinical information of HR‐positive, HER‐2 negative metastatic breast cancer patient treated with fulvestrant plus ribociclib. Possible adverse events such as elevated serum creatinine and lactic acidosis were observed and discussed with the current knowledge of scientific evidence.

## 2 CASE REPORT

**FIGURE 1:**
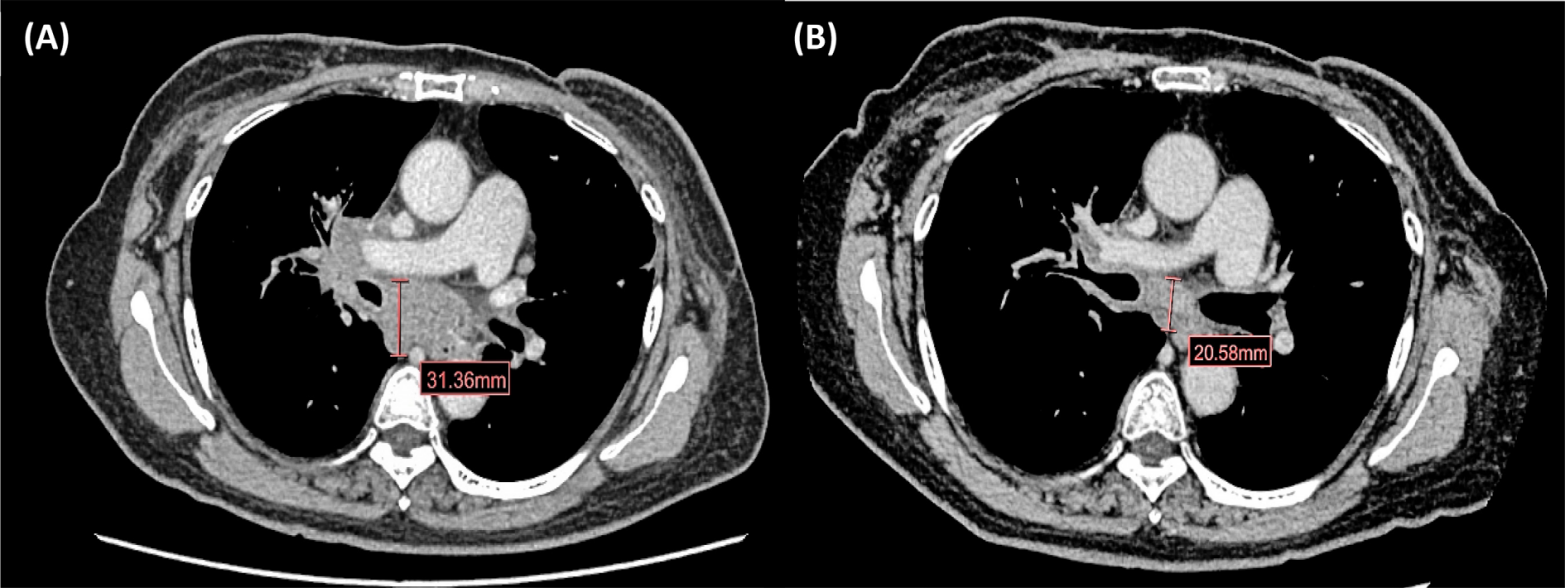
Chest computed tomography (CT) scan of target lesion during palbociclib plus fulvestrant. In April 2019, baseline chest CT scan before fulvestrant plus palbociclib was started (A); In September 2019, chest CT scan showed decreasing in size of mediastinal and hilar lymph nodes (chest CT scan before changing palbociclib to ribociclib plus fulvestrant according to Thai OCPA) (B)

A 62‐year‐old female with a history of NIDDM, dyslipidemia, and HT, previously was diagnosed with stage IIB left triple‐negative breast cancer in 2008. Left modified radical mastectomy and completed AC (doxorubicin and cyclophosphamide) followed by paclitaxel were given. Postoperative radiotherapy was performed after adjuvant chemotherapy. In December 2014, she developed a new right breast mass. She underwent right nipple‐sparing mastectomy with sentinel lymph node dissection with reconstruction. Rt breast pathology showed invasive ductal carcinoma, second primary breast cancer, pT1cN0M0, stage IA, (ER: 10%, PR: 10%, HER2‐negative, Ki67: 10%). Adjuvant TC (docetaxel and cyclophosphamide) for 4 cycles followed by tamoxifen was administered since July 2015. In February 2018, she developed chronic cough with weight loss for 5 kg. chest computed tomography (CT) scan showed mediastinal, right hilar, right interlobar lymph nodes metastases with multiple discrete lung nodules. The liver and bone were unremarkable. Mediastinal lymph node biopsy at 11R and 4R station was performed and reported invasive ductal carcinoma (ER: 60%, PR: negative, HER‐2 2+, and negative by ISH testing). She was then diagnosed with recurrent HR positive HER2 negative breast cancer. Due to her cough with dyspnea, palliative chemotherapy, docetaxel was given for eight cycles with partial response. Letrozole was then maintained for 9 months from August 2018 to May 2019. Because of disease progression with no visceral crisis and reimbursement policy, a second‐line combination of fulvestrant and palbociclib has been subsequently started since May 2019. Neither neutropenia nor GI toxicity was observed during this treatment. The following chest CT scan for evaluation was performed and showed a partial response as shown in Figure 1. She regularly took metformin 2000 mg/day, glipizide 5 mg/day, losartan 100 mg/day, amlodipine 10 mg/day, and simvastatin 10 mg/day since May 2019. Her serum creatinine was stable approximately 0.9–1.07 mg/dl during palbociclib treatment.

**FIGURE 2:**
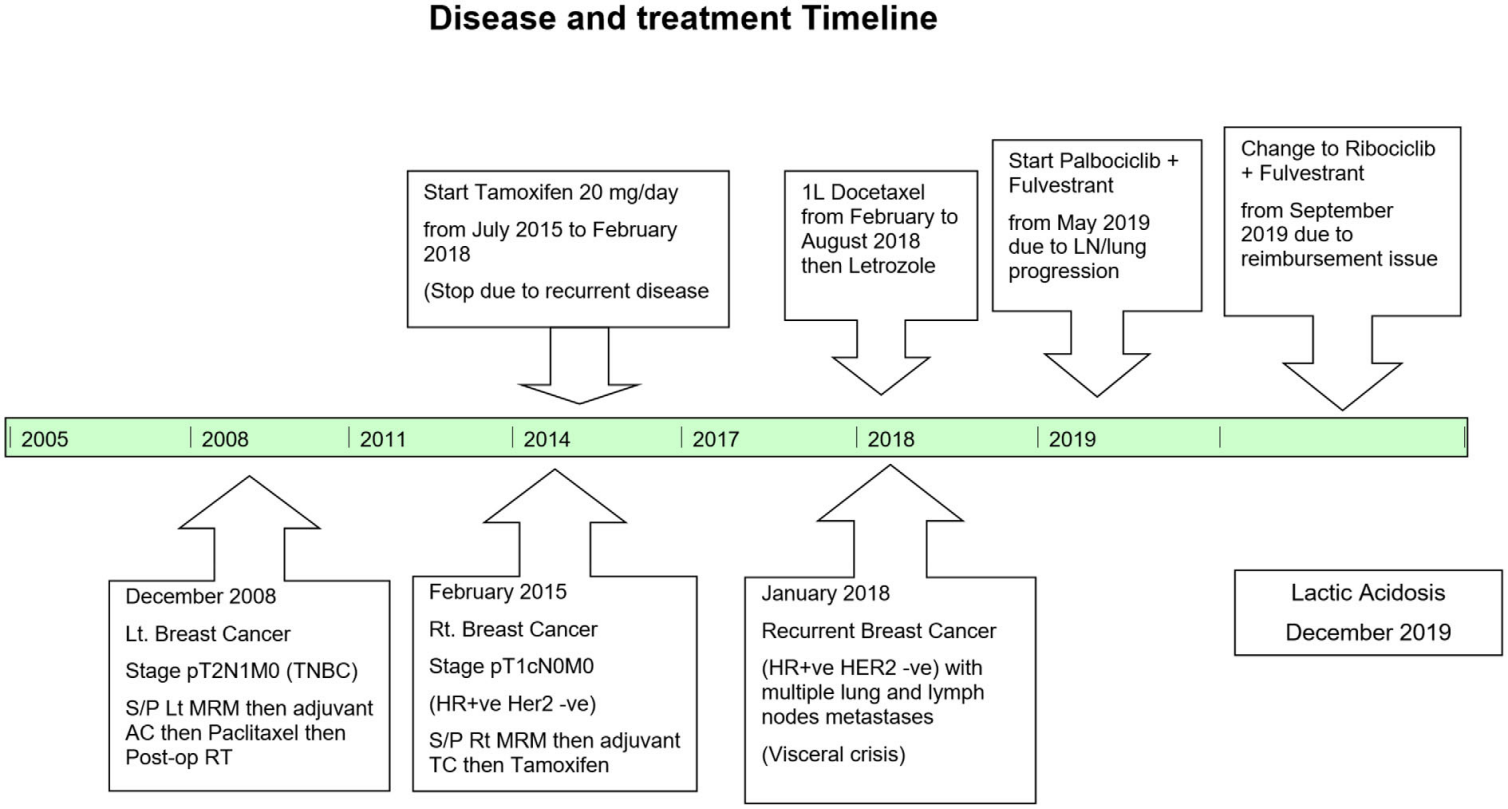
Summary disease and treatment timeline

In September 2019, due to the Thai Oncology Prior Authorization (OCPA) program, the patient had been requested to change treatment from palbociclib to ribociclib (600 mg/day) plus fulvestrant. Otherwise, she cannot do reimbursement for palbociclib's cost. During follow up, occasional mild palpitation without QTc prolongation was reported. Neither neutropenia nor GI toxicity was observed during this treatment like previous palbociclib. However, approximately 4 weeks after changing to ribociclib, her serum creatinine gradually increased from 0.94 to 1.31 mg/dl. She currently took antidiabetic, antihypertensive and antidyslipidemic drugs without dose modification. Moreover, no herbal products or oral supplements or NSAIDs were reviewed. Her disease and treatment timeline were shown in Figure 2.

**FIGURE 3:**
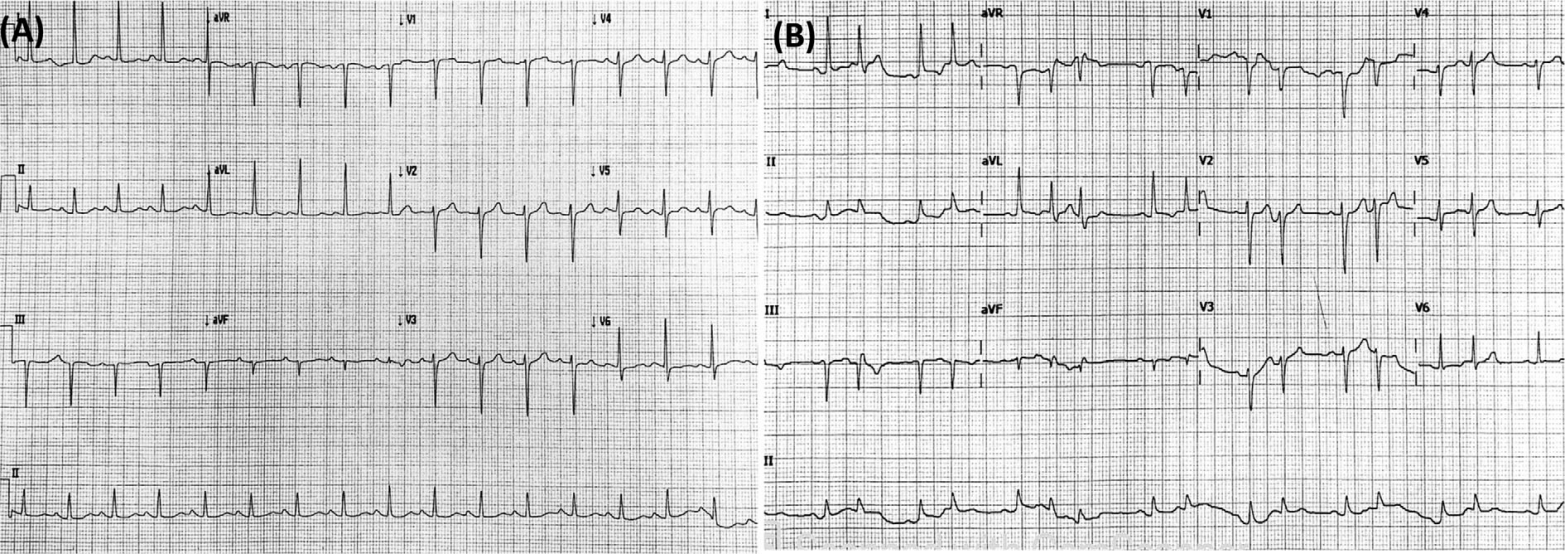
Electrocardiograms (ECGs) 12‐lead during treatment with ribociclib. In September 2019 (prior ribociclib initiation), QTcF (Fridericia's correction formula for prolongation of QT interval) was 406 ms (A); In November 2019 at ER, ECG develops new‐onset arrhythmia with 423 ms QTcF (B)

In November 2019, she was sent to the emergency department with progressive dyspnea for 1 week with intermittent epigastric pain, nausea but no vomiting. On admission, she had blood pressure 124/65 mmHg, pulse 106 beats per minute, respiratory rate 22 times per minute, and core body temperature 36.3°C. Initial investigations showed blood urea nitrogen 56 mg/dl, creatinine 2.8 mg/dl, sodium 133 mEq/L, potassium 5.6 mEq/L, chloride 102 mEq/L, bicarbonate 6 mEq/L, CPK 715 U/L (29–168 U/L), glucose 217 mg/dl and blood lactate 13.7 mmol/L (<2 mmol/L). Arterial blood gas showed severe mix wide anion gap acidosis plus normal gap metabolic acidosis (pH 7.12, pCO_2_ 20.8 mmHg, Po_2_ 66.8 mmHg, anion gap 25). Complete blood count indicated leukocytosis with left shift (WBC 21320/mm^3^, N 94%, L2.9%). The 12‐leads electrocardiogram (ECG) was performed in the emergency room as shown in Figure 3.

**FIGURE 4:**
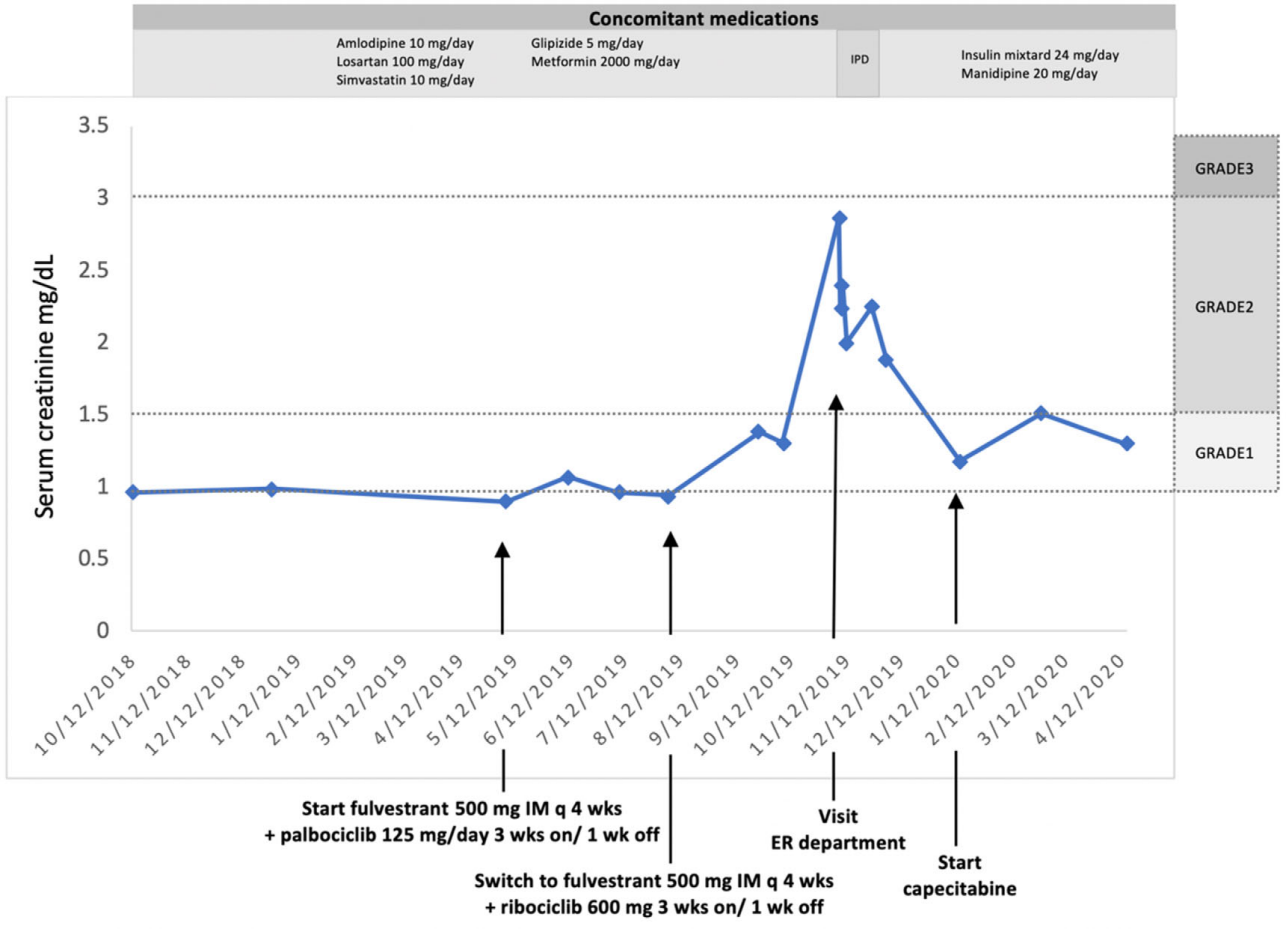
Changes of serum creatinine between October 2018 and April 2020. GRADE: Grade of serum creatinine increase according to Common Terminology Criteria for Adverse Events version.5.0; IPD, inpatient department; ER, emergency department

This patient was diagnosed with severe lactic acidosis, suspected from metformin‐associated with preexisting renal insufficiency with acute respiratory failure. Ribociclib and fulvestrant were temporarily discontinued. Three days after renal replacement therapy, her clinical was stabilized; she was able to extubate and remove double lumen. Her renal function gradually recovered, and serum creatinine was reduced to 2.25 mg/dl. Additionally, her creatinine further decreased to 1.51 mg/dl in 1 month after discharge to the hospital. All changes in serum creatinine were shown in Figure 4. Drug–drug interactions from ribociclib with metformin have been reviewed previously. So, the potential reason for severe lactic acidosis is considered to be from metformin associated lactic acidosis (MALA) predisposing from pre‐renal acute kidney injury with drug–drug interaction from ribociclib with metformin.

Owing to her severe renal impairment persist after discharge, permanent withholding ribociclib was continued but fulvestrant monotherapy was given since December 2020. At that time, her chest CT scan in January 2020 showed progressive disease at lymph nodes, lung, and liver nodules. Fulvestrant was stopped and then capecitabine was given. Currently, she continuously received capecitabine with good performance status and response. No adverse event was observed.

## 3 DISCUSSION

Here, we demonstrate potential lethal toxicity from co‐administration of ribociclib, metformin, losartan, and amlodipine with simvastatin in advanced breast cancer patients who had preexisting renal impairment. However, no special adverse events have been identified during palbociclib treatment. Renal impairment was detected in 4 weeks after ribociclib initiation. Then, severe metabolic acidosis, lactic acidosis (blood lactate level 13.7 mmol/L) unfortunately occurred after 8 weeks of administration. After renal replacement therapy and drug interruption, she gradually recovered. Her serum creatinine turned to her baseline level previously.

In drug–drug interactions (DDIs), both palbociclib and ribociclib are substrates of the CYP3A4 enzymatic complex.^6^ The co‐administration of palbociclib or ribociclib with strong or moderate CYP3A4 inhibitors or inducers may be either leading a risk of increased toxicity or diminished efficacy. Slightly different in the CYP3A4 enzymatic complex between two drugs, co‐administration with standard dose ribociclib may increase plasma concentrations of CYP3A4 substrates and accounts for more toxicities than palbociclib. Consistent with our report, no adverse event was observed during the administration of palbociclib. In contrast, elevation of serum creatinine and mild GI toxicities were reported a few weeks after the administration of ribociclib. Simvastatin, losartan, and amlodipine are the major CYP3A4 substrates. Taking ribociclib with these drugs affects drug metabolisms which subsequence develops toxicities as gradual renal impairment. Previously, concomitant simvastatin with palbociclib showed statin‐induced rhabdomyolysis in a case study.^11^ Changing ribociclib to palbociclib may report similar adverse reactions. According to effect in membrane transporters, both palbociclib and ribociclib can potentially inhibit different membrane transporters and different locations. However, unlike ribociclib, palbociclib has a low potential inhibition of OATP1B1, OATP1B3, BSEP, OAT1, OAT3, and OCT2.

Therefore, combination metformin with ribociclib can lead to the hypothetical inhibition of renal transporters which increase in metformin concentration and harmfulness which is higher frequency than combination with palbociclib.

Elevation of serum creatinine has been commonly recognized in many studies of abemaciclib.^2^, ^12^ Abemaciclib can inhibit renal efflux transporters, including MATE1 and MATE2‐K, thereby preventing the secretion of creatinine into the renal tubule. However, no impaired renal function, glomerular filtration rate, was established.^13^ Similarly, ribociclib may inhibit renal transporters for instance OCT2, MATE1, BCRP, and BSEP at clinically relevant concentrations which lead to serum creatinine rising.^14^, ^15^ Additionally, close monitoring of creatinine levels in patients who have impaired renal function is critical. Unlike MONALEESA‐2^16^ and MONALEESA‐7 study,^17^ the elevation of serum creatinine was recognized in MONALEESA‐3.^4^, ^5^ The incidence of all grades and grade 3–5 of renal toxicity in patients who received ribociclib plus fulvestrant was 12.6% and 1.4%, respectively.

**FIGURE 5:**
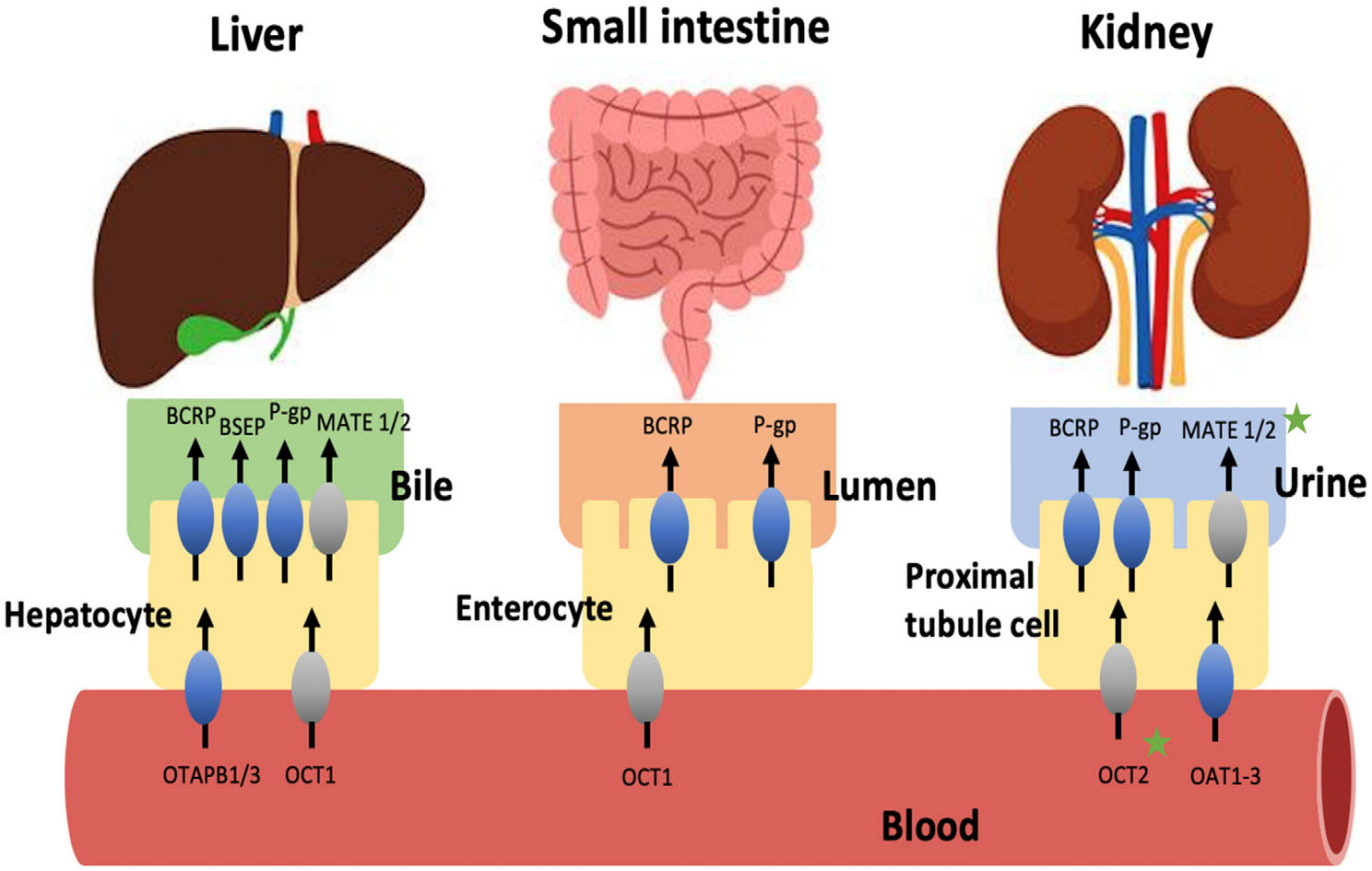
According to an in vitro study, ribociclib acts as inhibitors of various membrane transporters especially BRCP, OCT2, MATE1, and BSEP. Metformin is a drug substrate demonstrated in the gray color transporter. However, only OCT2 and MATE1 play potential role of DDI between ribociclib and metformin demonstrated in green star. As a result, a greater amount of metformin would accumulate in the blood causing the appearance of adverse effects. ABC, ATP‐binding cassette; BCRP, breast cancer resistance protein; BSEP, bile salt export pump; MATE1, multidrug and toxin extrusion protein; OATP, organic anion‐transporting polypeptide; OCT, organic cationic transporter; P‐gp, P‐glycoprotein; Adapted from Ther Adv Med Oncol 2019, Vol. 11: 1–43.^8^, ^9^, ^10^

Lactic acidosis arises from the accumulation of blood lactate and protons in the body fluids and is related to cellular dysfunction and poor clinical outcomes. Disorders associated with tissue hypoxia from sepsis, cardiogenic or hypovolemic shock, and heart failure are the major causes of this problem. Furthermore, cancer itself and several drugs including metformin can trigger lactic acidosis. Metformin, a widely used oral antidiabetic drug in the biguanide class, is excreted unchanged via active tubular secretion by MATE1/2 and OCT2 in urine.^18^ Under therapeutic conditions, ribociclib has a potential to inhibit OCT2, MATE1, BCRP, and BSEP. Hence, administration of metformin concomitant with ribociclib results in the increased exposure to metformin and a decrease in metformin renal clearance.^8^ Ribociclib acts as inhibitors of various membrane transporters as shown in Figure 5. In our study, even though the plasma concentration of metformin was not evaluated, in the patient who had preexisting renal impairment, high plasma metformin levels well recognized and prompted initiating of MALA.

Clinical presentations of MALA are nonspecific and sometimes life threatening. Hypotension, hypothermia, cardiac dysrhythmias, and respiratory failure requiring mechanical ventilation have been reported. After metformin discontinuation, adequate supportive care includes mechanical respiratory support in patients with respiratory distress, assurance of suitable systemic perfusion, correction of fluid deficits and electrolyte abnormalities, treatment of hypothermia and hypoglycemia are suggested.^19^ Other identified causes must also be effectively managed. Since metformin has a large distribution volume and slow rate of elimination from the deep compartment, long and multiple sessions of hemodialysis are necessary for preventing the rebound lactic acidosis in severe cases.

In resuming treatment with ribociclib, no available evidence supports using this drug in severe renal impairment (CrCl <30 ml/min). However, only a small proportion of ribociclib is eliminated by the renal route.^9^, ^20^ Ongoing study of ribociclib in healthy patients with various degrees of renal impairment is currently recruiting participants and waiting for good outcomes.

## 4 CONCLUSION

Lactic acidosis may occur after the coadministration of ribociclib and metformin in patients with pre‐existing renal impairment. Change in metformin pharmacokinetics from drug–drug interactions with CDK4/6 inhibitors should be monitored cautiously especially in patients with preexisting renal impairment.

## HUMAN AND ANIMAL RIGHTS

No animals were used in this research. All research procedures were in accordance with the Helsinki Declaration of 1975, as revised in 2008.

## CONSENT FOR PUBLICATION

The study has been approved by Institutional Ethical Committee. Informed consent for publication was obtained from the patient.

## CONFLICT OF INTEREST

The authors have stated explicitly that there are no conflicts of interest in connection with this article.

## AUTHOR CONTRIBUTIONS

All authors had full access to the data in the study and take responsibility for the integrity of the data and the accuracy of the data analysis. CL: Data curation, investigation, methodology, formal analysis, writing‐original draft, C.L.; Data curation, investigation, supervision, validation, N.Po.; Data curation, investigation, project administration, methodology, supervision, validation, N.Pa.

## Data Availability

The Authors allow the Journal to share all the published data, although there is not an online repository which they have been collected in.
